# Acute changes in blood lipid profiles and metabolic risk factors in collegiate elite taekwondo athletes after short-term de-training: a prospective insight for athletic health management

**DOI:** 10.1186/s12944-017-0534-2

**Published:** 2017-07-24

**Authors:** Yu-Chi Sung, Yi-Hung Liao, Chung-Yu Chen, Yu-Liang Chen, Chun-Chung Chou

**Affiliations:** 1 Department of Chinese Martial Arts, Chinese Culture University, Taipei City, Taiwan; 2 Department of Exercise and Health Science, National Taipei University of Nursing and Health Sciences, Taipei City, Taiwan; 3 Department of Exercise and Health Sciences, University of Taipei, Taipei City, Taiwan; 4 Graduate Institute of Coaching Science, Chinese Culture University, Taipei, Taiwan; 5 Physical Education Office, National Taipei University of Technology, 1, Sec. 3, Zhongxiao E. Rd, Taipei City, 10608 Taiwan

**Keywords:** Body composition, Atherogenic dyslipidemia index, Platelets, McAuley’s index, Baseline inflammation

## Abstract

**Background:**

This study used a short-term de-training model to mimic the physiological weight changes during the early retirement stage in Taekwondo (TKD) athletes. This study investigates whether the negative changes in body composition, blood lipid profiles, and metabolic biomarkers occur in elite collegiate TKD athletes when experiencing a two-months de-training period.

**Methods:**

Fourteen collegiate Division Ι elite TKD athletes (age: 21.1 ± 0.2 years, BMI: 22.3 ± 1.1 kg/m^2^; 10 males and 4 females) participated in this study. The body composition, blood lipid profiles, atherogenic dyslipidemia indexes, metabolic biomarkers and baseline systemic inflammation states were measured before and after two-months de-training.

**Results:**

The body weight and BMI did not change after de-training in these elite TKD athletes. The total muscle mass displayed a significant decline after de-training (−2.0%, *p* = 0.019), with an increase in fat mass (+24.3%, *p* < 0.01). The blood triglyceride did not change, but the total cholesterol was higher after de-training (+8.3%, *p = 0.047*). The CHOL-to-HDL and LDL-to-HDL ratios increased by 12.4% (*p* < 0.001) and 13.2% (*p* = 0.002) after de-training, respectively. The blood platelet number, plateletcrit, and platelet-to-lymphocyte ratio increased significantly by 5.0% (*p = 0.013*), 7.3% (*p = 0.009*), and 20.6% (*p = 0.018*) after de-training, respectively. The McAuley’s Index decreased (−6.9%, *p = 0.025*) after de-training.

**Conclusion:**

We demonstrated that a two-months de-training period resulted in adverse effects on early atherogenic dyslipidemia development, progressing insulin resistance, low-grade inflammation, and visceral adiposity in young elite TKD athletes. Our findings provide clear insights into the possible deleterious impacts at early stage retirement in former combative sports athletes.

## Background

Competitive athletes in certain types of sports, such as basketball, tennis, running, and swimming etc., may maintain the same type of exercise for recreational purposes during their off-season period or after retirement. Conversely, in combat sports athletes (e.g. taekwondo, judo, karate, etc.), the competitive fighting pattern is difficult to convert to their daily recreational activity style. The discontinuance of regular physical activity or training has been demonstrated to increase the risk for cardiovascular diseases, such as the negative change in obesity development, dyslipidemia and metabolic disorders [1–5]. Moreover, previous evidence has revealed that the dramatic decrease in physical activity level may be more susceptible to inactivity-related increases in disease risk in retired athletes [6–8]. Therefore, for former competitive athletes who train for many years to reach the elite level should notice a dramatic decrease in the amount of physical activity after retirement. However, there is very limited cohort follow-up scientific evidence for tracing the metabolic degenerations and/or better managing the cardiovascular health in these retired elite athletes. Studies involving de-training models appear to be appropriate for mimicking the early stage of retirement in these former combative sports fighters, despite only several recent studies investigating the effects of de-training on the declines in sport-specific performance [9, 10].

Taekwondo (TKD), a traditional Korean martial art, is an official Olympic sports discipline. The number of TKD practitioners has grown around the world [11]. Similar to most combat sports, TKD athletes should compete according to weight category, and most TKD athletes experienced fluctuations in body weight in their athletic career. Study evidence has shown that the fluctuations in body mass may be one of the primary risk factors for the development of obesity, diabetes and cardiovascular disease in later life [12]. The association between weight fluctuations and future cardiovascular morbidity and mortality has been clinically demonstrated [13], and the negative alterations in blood lipid profiles can have a strong deleterious influence on future cardiovascular events [14]. However, the acute changes in these health-related blood biomarkers in elite Taekwondo athletes after short-term de-training have not been evident in the current literature.

In this regard, we hypothesized that after a short-term interruption of intensified TKD-specific training, the body composition, blood lipid profiles and metabolic regulatory capacity would present rapid negative changes in these young elite collegiate TKD combat fighters. Therefore, the purpose of this study is to investigate whether the acutely negative changes in these parameters occur in elite collegiate TKD athletes when experiencing a two-months de-training period.

## Methods

### Participants

Fourteen collegiate Division Ι elite TKD athletes (age: 21.1 ± 0.2 years, weight: 68.3 ± 3.7 kg, height: 174.6 ± 1.9 cm, BMI: 22.3 ± 1.1 kg/m^2^; 10 males and 4 females) volunteered to participate in this study. These TKD combative sport fighters were all black belt holders with at least 6 years competition experience. The participating TKD athletes were all active in either national or international level competitions. Several of these participants were selected for the Taiwanese national team for the Asian Taekwondo Championship events. The competing category for these collegiate participants were classified in accordance with the International University Sports Federation (FISU) rules, including ten males: <54 kg (*n* = 1), 58–63 kg (*n* = 2), 63–68 kg (*n* = 2), 68–74 kg (*n* = 2), 74–80 kg (*n* = 2), 80–87 kg (*n* = 1); and four females: 46–49 kg (*n* = 1), 53–57 kg (*n* = 1), >73 kg (*n* = 2). The experimental procedures were reviewed and approved by the Institute Review Board of the University of Taipei before the participant’s recruitment began. All procedures were conducted in accordance with the guidelines provided by the IRB and the principles expressed in the Declaration of Helsinki. The experimental purpose and procedures were explained to all participants. Participants completed a written informed consent before the experiment. All participants were free of cardiovascular diseases, hypertension, musculoskeletal sports injuries, diabetes and metabolic disorders.

### Experimental design and procedure

This study was conducted to test the hypothesis that a two-month de-training period would result in dramatic deleterious effects on body composition, blood lipid profiles, risk factors for future cardiovascular events, metabolic functions and baseline inflammation states in elite collegiate Taekwondo athletes. Measurements during this investigation were performed during 3 distinct phases: (a) competition preparation season with intense TKD training (measurements were conducted two weeks before the end of the season), (b) during 2-month de-training (just during the summer vacation for college students), and (c) after two months of de-training. During the competition preparation season, the regular collegiate TKD weekly training program (5 days/week) was organized in a form with three days of high-intensity training (strength/condition training plus TKD-skill/tactical training) and two days of low-intensity training (TKD-skill training plus opponent video evaluation by coach). The body composition measurements and venous blood sampling were performed two weeks before the end of the competition season/semester. Only light walking and daily required activities were allowed at least two days before the overnight fasting (12 h) venous blood sample collection to minimize the possible confounding factors for the metabolic biomarker measurements. Afterward, the participants were instructed to only perform light-moderate physical activity (<6.0 METS) and daily required activities throughout the entire de-training period. They were also directed to maintain their regular TKD-training dietary intake patterns to minimize the possible factors from dietary changes during the 2-month de-training. For the dietary control purpose, we provided a general dietary guideline, which containing the recommended types and amount of food to consume during the period of detraining (e.g. carbohydrate: ~60%; protein: 10–15%; and fat:15–25%), according to their estimated basic energy expenditure. During the de-training period the Taekwondo-specific training and strength/conditioning training were completely interrupted for two months. At the end of de-training all measurements, including body composition and venous blood sample collection, were performed to compare the changes in blood lipid profiles and metabolic functions after a short-term interruption of intensified TKD-specific training.

### Taekwondo athletic exercise training procedure

The TKD-specific training program during the competition season was designed based on the requirement for preparing national collegiate championship. In brief, this TKD weekly training program (5 days/week; 10 training sessions/week; totally ~14–15 h/week) consisted of three days of high-intensity training (strength/condition training plus TKD-skill/tactical training; performed on Monday, Wednesday, and Friday) and two days of low-intensity training (TKD-skill training plus opponent video evaluation by coaches; performed on Tuesday and Thursday). In a week-based training regimen, the strength and conditioning training included (i) 2–2.5 h of aerobic and anaerobic energy system training (jogging, fast running, shuttle running, and sprinting; intensities were set between 65 and 85% maximum heart rate), (ii) 2.5 h of resistance training (movements consisting of quadriceps extension, hamstring curl, leg press, standing leg curls, and bench press; intensities were set between 65 and 80% 1 repetition maximum), and (iii) 2 h of stretch, agility, and flexibility training. The weekly TKD-specific tactical and skill training included (i) 5.5–6 h of attacking skill training (punching, kicking, side-kicking, spin-kicking, step/feint motions, etc.), (ii) 1–1.5 h of simulated match training, and (iii) 0.5–1 h of opponent video evaluation (coaches instructed attacking strategies and fighting tactics). This training program was conducted until the end of the competition season/semester, thereafter all the specific training activities stated above were refrained from in their regular daily activity during the de-training period.

### Body composition

The body composition was determined using a bioimpedance body composition analyzer (*InBody 720,* InBody Co., Ltd., Seoul, Korea). The measurement was performed after overnight fasting (12 h). The drinking of liquids was prohibited at least 4 h before the body composition assessment in accordance with the manufacturer’s instructions. The obtained values were used to determine the muscle mass, fat mass and estimated visceral fat area [15].

### Analyses of platelet-related profiles, serum insulin, and blood lipid profiles

The EDTA-treated whole blood samples were used to determine platelet-related profiles, including blood platelet number (PLA), mean platelet volume (MPV), and platelet crit (PCT) using a hematology analyzer (Sysmex XT-2000, Sysmex Corp., Kobe, Japan). Whole blood samples collected in red-cap tubes with clot activator (Molecular Diagnostic Services, Inc., San Diego, CA, USA) were centrifuged at 3000×g for 10 min (4 °C). The supernatant was then collected and stored at −80 °C for later circulating insulin and blood lipid profile analysis. The fasting blood lipid profiles, including triglyceride, total *cholesterol (CHOL), LDL cholesterol (LDL),* and *HDL cholesterol* (*HDL)* were measured using UniCel DxC-800 Synchron Clinical Systems (Beckman Coulter, Inc., Brea, CA, USA). Serum insulin concentration measurement was performed using the enzyme-linked immunosorbent assay method (ELISA) with commercially available kits used to determine the insulin serum levels (intra-assay CV% = 3.35%; #10–1113-01, Mercodia AB, Uppsala, Sweden) in accordance with the manufacturers’ instructions.

### Measurements of insulin resistance

The insulin resistance assessment was determined using McAuley’s index (a triglyceride- and insulin-based method), which was widely used in larger studies and at times in the clinical setting [16]. The McAuley’s index presents high correlation with the insulin resistance measurements from gold-standard techniques, and this biomarker is appropriate to evaluate the level of insulin resistance irrespective of age, sex, renal allograft function, and obesity [16]. This index was calculated using insulin and triglyceride at fasting levels, and the formula of McAuley’s index = [2.63–0.28 × ln insulin (mU/L) - 0.31 × ln triglycerides (mmol/L)].

### Statistical analysis

All data were expressed as mean ± standard error of mean (Mean ± S.E.M.), and the percent changes (Δ% between pre- and post-de-training) in the measured parameters were calculated using the formula = [(post-de-training value - pre-de-training value)/(pre-de-training value)] × 100%. The normality of all the data were determined by using Shapiro-Wilk normality test in prior to performing the t-test. There was one variable (i.e. HDL/LDL ratio) showing skewed distribution of the data. Thereafter, this variable was then logarithmically transformed for the later *paired t-test* statistical comparison when the normality of data presented. All measurements were analyzed using a paired *t-test* to compare before and after eight-week de-training, and the *p* ≦ 0.05 was used for determining statistical significance. The statistical analysis and data graphing were performed using a commercialized statistical software package (SPSS 16.0, IBM Inc., Chicago, IL, USA) and GraphPad Prism 5.0 (GraphPad software Inc., La Jolla, CA, USA).

## Results

### Changes in body composition profiles before and after de-training

The body composition profiles measured using bioimpedance method are displayed in Table 1. The body weight and BMI did not change after two-months de-training in these elite TKD athletes. Total muscle mass displayed a significant decline after de-training (−2.0 ± 0.9%, *p* = 0.019). There were no changes in muscle mass in both the arms and trunk, but the decreased muscle mass from both legs primarily contributed to the overall loss in skeletal muscle (left leg: −2.0 ± 0.8%, *p* = 0.020; right leg: −2.1 ± 0.8%, *p* = 0.016). The fat mass increased significantly from 12.3 *kg* to 14.3 *kg* (+ 24.3%, *p* < 0.01), and the visceral fat area increased significantly from 42.8 *cm* ^*2*^ to 56.4 *cm* ^*2*^ (+ 37.3%, *p* < 0.001).

**Table 1:** Body composition profiles at before and after two-months detraining in elite collegiate Taekwondo athletes (*n* = 14; 10 males and 4 females)

	Body Composition Profiles (*n* = 14; 10 males and 4 females)				
	TKD Training Period	Two-months Detraining	Pre-Post % change	*P* value	Sig.
Weight (kg)	68.3 ± 3.7	69.5 ± 3.5	2.0 ± 1.2%	0.082	*n.s.*
BMI (kg/m^2^)	22.3 ± 1.1	22.7 ± 1.1	2.0 ± 1.2%	0.086	*n.s.*
*Skeletal Muscle Tissue*					
Total muscle mass (kg)	31.6 ± 1.4	31.0 ± 1.5	−2.0 ± 0.9%	0.019	*
Left arm (kg)	2.8 ± 0.2	2.8 ± 0.2	−0.2 ± 1.2%	0.475	*n.s.*
Right arm (kg)	2.8 ± 0.2	2.8 ± 0.2	−0.7 ± 1.4%	0.356	*n.s.*
Trunk (kg)	23.6 ± 1.0	23.6 ± 1.0	−0.3 ± 0.8%	0.423	*n.s.*
Left leg (kg)	9.1 ± 0.4	9.0 ± 0.4	−2.0 ± 0.8%	0.020	*
Right leg (kg)	9.2 ± 0.4	9.0 ± 0.4	−2.1 ± 0.8%	0.016	*
*Adipose Tissue*					
Total body fat mass (kg)	12.3 ± 2.5	14.3 ± 2.3	24.3 ± 7.3%	0.004	**
Visceral fat area (cm^2^)	42.8 ± 5.6	56.4 ± 6.8	37.3 ± 7.3%	<0.001	***

*TKD* Taekwondo, *BMI* body mass index, *Sig.* significance, *n.s.* non-significant difference. All data were expressed as Mean ± S.E.M. * denotes the significant difference between pre- and post-detraining (*p* < 0.05). ** denotes the significant difference between pre- and post-detraining (*p* < 0.01). *** denotes the significant difference between pre- and post-detraining (*p* < 0.001)

### Blood lipid profiles and atherogenic dyslipidemia indeses

**Fig. 1.**
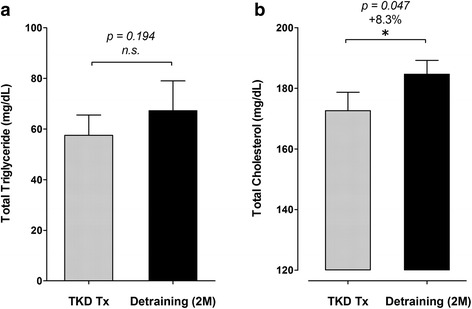
Changes in triglyceride (TG) and total cholesterol (CHOL) at before and after a two-months de-training in the elite TKD collegiate athletes. The circulating levls of (**a**) triglyceride (TG; mg/dL) and (**b**) total circulating cholesterol (CHOL; mg/dL) were measured during regular competition training/preparation season (TKD Tx; *gray bar*) and after two-months de-training (De-training [2 M]). All values are expressed as *Mean ± S.E*.*M.* * denotes the significant difference between TKD Tx and De-training [2 M] (*p* < 0.05).

**Fig. 2:**
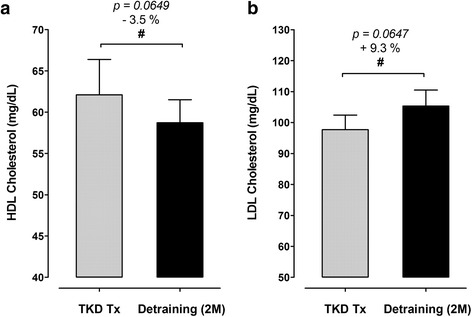
Changes in high-density lipoprotein cholesterol (HDL) and low-density lipoprotein cholesterol (LDL) at before and after a two-months de-training in the elite TKD collegiate athletes. The circulating levls of **a** high-density lipoprotein cholesterol (HDL; mg/dL) and **b** low-density lipoprotein cholesterol (LDL; mg/dL) were measured during regular competition training/preparation season (TKD Tx; *gray bar*) and after two-months de-training (De-training [2 M]). All values are expressed as *Mean ± S.E*.*M.* # denotes the approached significant difference between TKD Tx and De-training [2 M] (*p < 0.075*)

**Fig. 3:**
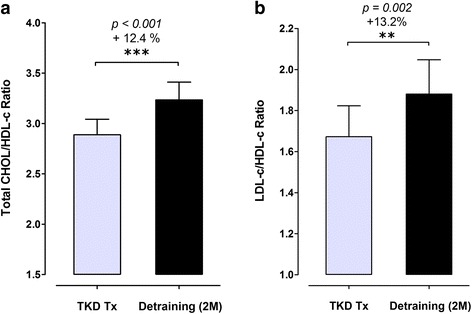
Changes in atherogenic dyslipidemia indexes before and after a two-months de-training period in elite TKD collegiate athletes. The atherogenic dyslipidemia indexes, including **a** the ratio of total cholesterol-to-low-density lipoprotein cholesterol (CHOL/LDL ratio) and **b** the ratio of low-density lipoprotein cholesterol-to-high-density lipoprotein cholesterol (LDL/HDL ratio) were measured during regular competition training/preparation season (TKD Tx; *gray bar*) and after two-months de-training (De-training [2 M]). All values are expressed as *Mean ± S.E*.*M.* ** denotes the approached significant difference between TKD Tx and De-training [2 M] (*p < 0.01*); *** denotes the approached significant difference between TKD Tx and De-training [2 M] (*p < 0.001*)

Figures 1a and b display the changes in blood triglyceride (TG) and total cholesterol (CHOL) in response to a two-months de-training in the elite TKD collegiate athletes, respectively. There were no differences in blood TG level between TKD-specific training period (TKD Tx) and after de-training (De-training (2 M)), but the CHOL level was significantly higher after two-months de-training (+8.3%, *p = 0.047*). The differences in high-intensity lipoprotein cholesterol (HDL; −3.5%, *p = 0.0649*) and low-intensity lipoprotein cholesterol (LDL; +9.3%, *p = 0.0647*) were both approaching significant level between TKD training period and after de-training (Figs. 2a and b). The atherogenic dyslipidemia indexes (CHOL-to-HDL ratio and LDL-to-HDL ratio) are showed in Figs. 3a and b. Compared with the baseline at the TKD-specific training season, the total CHOL-to-HDL ratio and LDL-to-HDL ratio significantly increased by 12.4% (*p* < 0.001) and 13.2% (*p* = 0.002) after two-months de-training, respectively.

### Circulating platelets number and plateletcrit (PCT)

**Fig. 4:**
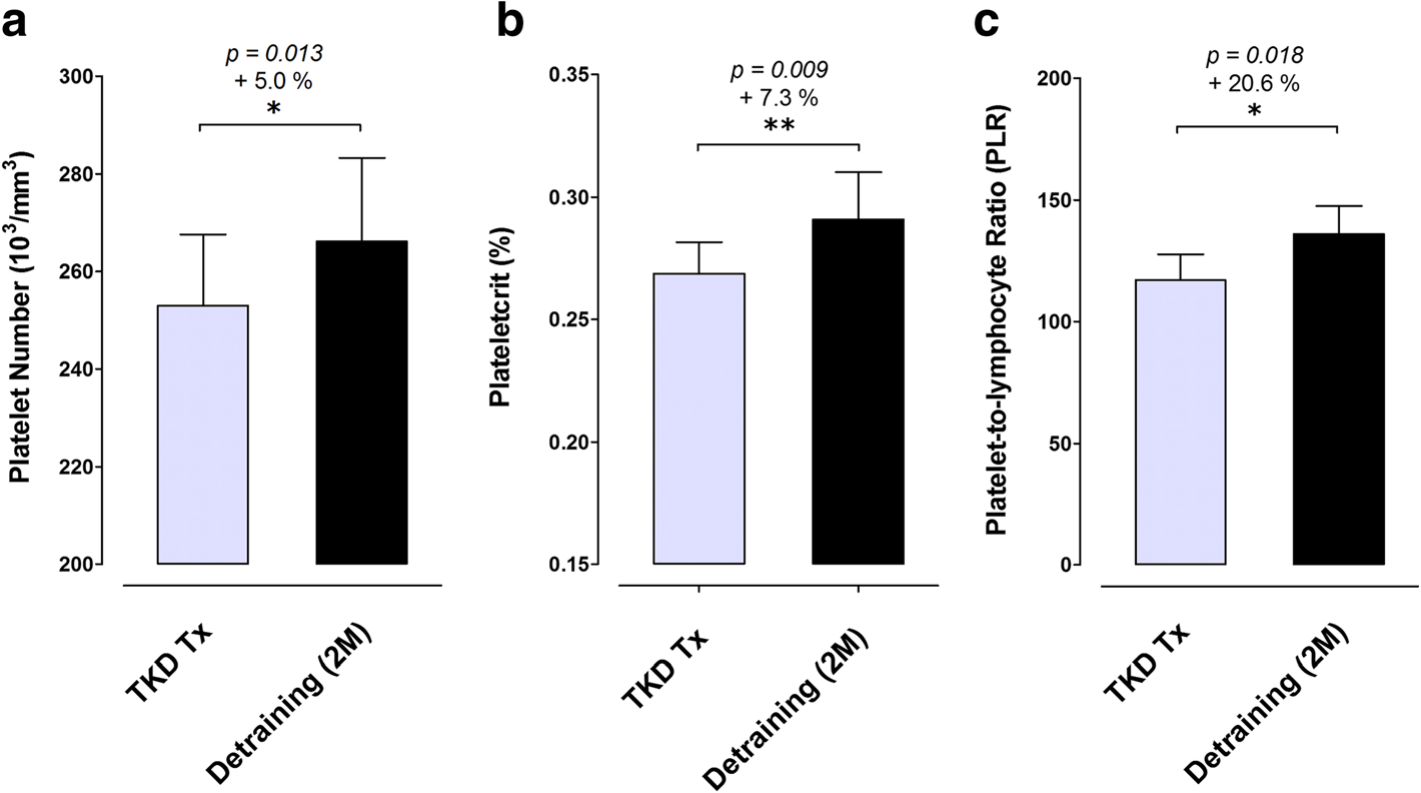
Changes in blood platelet number and plateletcrit at before and after a two-months de-training period in elite TKD collegiate athletes. The circulating levls of **a** platelet number (10^3^/mm^3^), **b** plateletcrit (PCT; %), and **c** platelet-to-lymphocyte ratio (PLR) were measured during regular competition training/preparation season (TKD Tx; *gray bar*) and after two-months de-training (De-training [2 M]). All values are expressed as *Mean ± S.E*.*M.* * denotes the approached significant difference between TKD Tx and De-training [2 M] (*p < 0.05*); ** denotes the approached significant difference between TKD Tx and De-training [2 M] (*p < 0.01*)

Figures 4a–c displayed the changes in blood platelet number (PLA), plateletcrit (PCT), and platelet-to-lymphocyte ratio (PLR) after a two-months detrainig in the elite TKD collegiate athletes, respectively. The PLA number increased significantly from 253.0 ± 14.6 10^3^/mm^3^ to 266.1 ± 17.2 10^3^/mm^3^ (+ 5.0%, *p = 0.013*) after de-training. The PCT values also increased significantly from 0.27 ± 0.01% to 0.29 ± 0.02% (+ 7.3%, *p = 0.009*) after de-training. The PLR values also increased significantly from 117.0 ± 10.6 to 136.1 ± 11.4 (+ 20.6%, *p = 0.018*) after de-training.

### Insulin sensitivity assessment

**Fig. 5:**
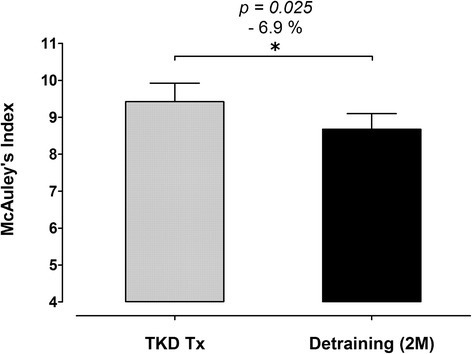
Changes in pherial insulin resistance status using McAuley’s Index at before and after a two-months de-training period in elite TKD collegiate athletes. The McAuley’s Index (based on the calculation using fasint insulin and TG levels) was measured and calculated during regular competition training/preparation season (TKD Tx; *gray bar*) and after two-months de-training (De-training [2 M]). All values are expressed as *Mean ± S.E*.*M.* * denotes the approached significant difference between TKD Tx and De-training [2 M] (*p < 0.05*)

The degree of insulin sensitivity was assessed using the McAuley’s Index, which was calculated based on the fasting insulin and triglyceride plasma levels. The fasting insulin levels were 9.9 ± 1.0 mU/L in the TKD-specific training period and 11.2 ± 1.0 mU/L after a two-months de-training (*p = 0.113*), and there was no difference between periods. The McAuley’s Index decreased significantly from 9.4 ± 0.5 to 8.7 ± 0.4 (− 6.9%, *p = 0.025*) after de-training, indicating that the insulin sensitivity was decreased in response to a dramatic decline in exercise training volume (Fig. 5).

## Discussion

The primary findings of this study were that two-months de-training resulted in adverse effects on body composition, blood lipid profiles, and the atherogenic dyslipidemia index in these elite collegiate TKD athletes. Moreover, the loss of leg muscle mass and the gain of visceral fat seem to be the predominant changes in body composition after de-training in these elite combat sport athletes. To our knowledge, we are the first to report on acute unfavorable changes in blood lipid profiles, development of future cardiovascular risks and insulin resistance development in elite young TKD athletes. We also provide the first available overview and evidence for changes in the risks for future metabolic and cardiovascular events after de-training in weight-categorized combat sport athletes. The evidence obtained in this study may provide new insight into health management in retired combative sports athletes.

There are a limited number of reports that have focused on the developing risks for metabolic disorders during the off-season in athletes, particularly in combative sports disciplines. Physical activity is a major contributor to reduce the prevalence of certain degenerative diseases and mortality [17]. The high-intensity physical activity level of these TKD athletes dramatically decreased by at least 14–15 h/week during the off-season compared with their regular training period. Our results reveal that the degree of insulin sensitivity was remarkably decreased by 6.9% (measured by McAuley-index) after 2-months de-training, indicating that insulin resistance had obviously developed. Because insulin resistance is one of the primary risk factors for several cardiovascular diseases (e.g. coronary artery and hypertensive diseases) [18], the rapidly rising insulin resistance may increase the risk for future metabolic disorders and cardiovascular events in these young elite athletes.

Previous evidence obtained from endurance athletes reveal that 3–8 weeks of insufficient physical activity would result in insulin resistance development [2, 3, 19], which is in line with our findings from retired combative athletes. On the other hand, the increase in circulating platelets has been documented in individuals with metabolic syndrome [20] and type 2 diabetes [21]. This biomarker is recognized as an independent predictor for insulin resistance. We also observed that 2-months de-training led to a slight but significant increase in blood platelet count by ~5.0% (from 253.0 to 266.1 10^3^/ mm^3^), and the level of central obesity also significantly increased in parallel (visceral fat area: from 42.8 to 56.4 cm^2^, +37.3%). These findings suggest that insulin resistance and the risks for future metabolic disorders develop rapidly in these young elite combat athletes [22–24].

Reduction in physical activity has adverse consequences on cardiovascular risk due in part to the detrimental effects of impaired lipid metabolism and serum lipoprotein concentrations [25, 26]. Lipid profile disorders and increases in the LDL-to-HDL ratio induced by long-term physical activity cessation (47 weeks) have been previously reported in high endurance-trained men [2]. Interestingly, retirement led to an increased circulating level of total CHOL (+8.3%), despite the decrease in HDL level and the increase in LDL after retirement showed an approaching significance (HDL: −3.5%, *p* = 0.065; LDL: +9.3%, *p* = 0.065) in these former elite collegiate TKD athletes. Furthermore, atherogenic dyslipidemia has also been reported in middle-aged men with long-term body weight fluctuations [27], and greater magnitudes of reported weight cycles being linked with significantly lower HDL [28] and increased risk for cardiac events [29].

Total CHOL, LDL, HDL, lipid ratio measurements are recommended in most current cardiovascular screening procedures [30]. Atherogenic dyslipidemia, characterized by the combinations of increased circulating levels of LDL, decreased HDL, and increased TG, has been recognized as an important risk factor for future myocardial infarction and cardiovascular disease development [31, 32]. Previous studies showed that the high LDL-C/HDL-C ratio was associated with hypertriglyceridemia and exhibited the highest coronary heart disease (CHD) risk [33, 34]. These findings suggest that the ratio of total CHOL/HDL-C can serve as an important cumulative index for the presence of atherosclerotic dyslipidemia and the risk of future CHD. During the off-season period, we observed that the indexes for atherogenic dyslipidemia, including total CHOL-to-HDL ratio and the LDL-to-HDL ratio, were substantially elevated after 8-weeks of de-training in these elite young TKD athletes. Because the LDL-to-HDL ratio [32] and total CHOL-to-HDL ratio [35] were demonstrated as appropriate biomarkers in the prediction of future cardiovascular events, our data reveals a clear trend for increased risks for future metabolic and cardiovascular events in these elite combat athletes. Together with these findings, our results, therefore, suggest that the risk for future cardiovascular events could be rapidly developed within 2-months of de-training in these elite collegiate combat athletes.

These atherogenic dyslipidemia and systemic inflammation developments are viewed as key risk factors for future cardiovascular events [31, 36]. Growing evidence reveals that the accumulation of adipose tissue leads to the development of systemic inflammation (e.g. increases in circulating levels of tumor necrosis factor-α and interleukin-6) [37, 38], which accounts for the obesity-associated vasculopathy and cardiovascular risks [39–41]. Recent studies indicate that plateletcrit (PCT), reflecting the total platelet mass, can serve as a surrogate biomarker for systemic inflammation [42] and increased CVD risk factors/events [43, 44]. Also, the platelet-lymphocyte ratio (PLR), another clinical used hematological inflammatory marker, has been recognized as a simple, inexpensive and rapid prognostic marker for future cardiovascular events [45]. Thus, we selected these two inflammatory markers to reflect the systemic inflammatory status in these young elite TKD athletes. Our results also showed that the visceral fat mass and systemic inflammatory status (reflected by ↑ PCT and ↑PLR) were significantly greater after 2-months de-training in these elite TKD athletes. Further, our data provides evidence that these young athletes showed rapid insulin resistance development within a short reduced training load period. We also observed a remarked increase in the atherogenic dyslipidemia indexes (↑ CHOL/HDL and ↑ LDL/HDL). Moreover, HDL-C is thought to have anti-inflammatory, anti-aggregatory, anticoagulant, and reverse cholesterol transport [46, 47], thus the decrease in HDL might account for the rapid systemic inflammation development. Although the decrease in HDL level after de-training showed an approaching significance in this study, we speculated that the HDL decrease trend in response to the reduced training load may still negatively affect the inflammation response and cardiovascular health.

According to our findings, the negative alterations in central obesity status, dyslipidemia development and metabolic fitness were observed after 2-months of de-training in these young elite collegiate TKD athletes. These impairments appear to be clearly associated with the dramatic declines in their sport-specific training volume. It has been well established that the higher fat mass, visceral fat accumulation, insulin resistance development, dyslipidemia and reduction in muscle mass are strongly associated with greater increases in cardiovascular risks in weight category athletes [12, 13]. We found these elite combat sports athletes possibly experienced more profound and faster changes toward metabolic disorder development in a relatively shorter period in compared with endurance athletes. The findings of this study are, therefore, important and likely to become a serious public health issue for these elite combat athletes. However, further researches are still needed to investigate the long-term changes in future cardiovascular diseases and metabolic disorders among these elite combat athletes. One of the limitations of this study is the limited sample size due to the population. The reason for the small number of subjects participated was that we needed to recruit elite TKD athletes to ensure the quality and well control of the study design. However, the qualified number of elite TKD athletes during recruitment were rare. Although the Type II error could not be completely excluded, future study with a large number of subjects would be required to investigate.

## Conclusions

This study demonstrated that a two-months de-training period was capable of creating adverse early atherogenic dyslipidemia development effects in young elite collegiate TKD athletes, progressing insulin resistance, low-grade inflammation, and visceral adiposity. Our findings that acute unfavorable changes in blood lipid profiles, the development of future cardiovascular risks and impaired metabolic functions provide clear insights into the possible deleterious impacts of early stage retirement in former combative sports athletes. The strengths of this study include its specific population-based design, reliable and detailed assessments of metabolic and cardiovascular risk factors. However, additional research with longer observation periods is still encouraged to determine the causal relationships among reduced physical activity, impaired lipid profiles, atherogenic dyslipidemia development and low-grade inflammation in former combat or weight class sports athletes.

## Abbreviations

CHOL: total cholesterol
ELISA: immunosorbent assay method
HDL: high density lipoprotein cholesterol
LDL: low density lipoprotein cholesterol
MPV: mean platelet volume
PCT: platelet crit (PCT)
PLA: blood platelet number
TG: triglyceride
TKD: Taekwondo
